# Insights of Nanostructured Ferberite as Photocatalyst, Growth Mechanism and Photodegradation Under H_2_O_2_-Assisted Sunlight

**DOI:** 10.3390/molecules30194026

**Published:** 2025-10-09

**Authors:** Andarair Gomes dos Santos, Yassine Elaadssi, Virginie Chevallier, Christine Leroux, Andre Luis Lopes-Moriyama, Madjid Arab

**Affiliations:** 1 Université de Toulon, Aix Marseille Univ, CNRS, IM2NP, 13397 Marseille, France; andarair@ufersa.edu.br (A.G.d.S.); yassine-elaadssi@etud.univ-tln.fr (Y.E.); cheva@univ-tln.fr (V.C.); christin.leroux@wanadoo.fr (C.L.); andre.moriyama@ufrn.br (A.L.L.-M.); 2 CCEN, UFERSA, Campus Mossoró-F. Mota, Costa e Silva, Universidade Federal Rural do Semi-Árido, Mossoró 59625-900, RN, Brazil; 3 Campus Universitário, L. Nova, Universidade Federal do Rio Grande do Norte, Natal 59072-970, RN, Brazil

**Keywords:** ferberite, photocatalysis, methylene blue degradation, hydrothermal synthesis, nanostructured materials, simulated sunlight irradiation, advanced water treatment

## Abstract

In this study, nanostructured ferberites (FeWO_4_) were synthesized via hydrothermal routes in an acidic medium. It was then investigated as an efficient photocatalyst for degrading organic dye molecules, with methylene blue (MB) as a model pollutant. The formation mechanism of ferberite revealed that the physical form of the precursor, FeSO_4_·7H_2_O, acts as a decisive factor in morphological evolution. Depending on whether it is in a solid or dilute solution form, two distinct nanostructures are produced: nanoplatelets and self-organized microspheres. Both structures are composed of stoichiometric FeWO_4_ (Fe: 49%, W: 51%) in a single monoclinic phase (space group P2/c:1) with high purity and crystallinity. The *p*-type semiconductor behavior was confirmed using Mott–Schottky model and the optical analysis, resulting in small band gap energies (≈1.7 eV) favoring visible absorption light. Photocatalytic tests under simulated solar irradiation revealed rapid and efficient degradation in less than 10 min under near-industrial conditions (pH 5). This was achieved using only a ferberite catalyst and a low concentration of H_2_O_2_ (4 mM) without additives, dopants, or artificial light sources. Advanced studies based on photocurrent measurements, trapping and stability tests were carried out to identify the main reactive species involved in the photocatalytic process and better understanding of photodegradation mechanisms. These results demonstrate the potential of nanostructured FeWO_4_ as a sustainable and effective photocatalyst for water purification applications.

## 1. Introduction

In the textile industry, approximately 800,000 tons of dyes are produced annually, with an estimated 15–20% being lost to wash water during the dyeing process. These dyes, predominantly synthetic, are characterized by complex molecular structures, high water solubility, and strong chemical stability, making them difficult to remove through conventional water treatment methods [1,2,3,4]. Traditional approaches include physical, chemical, and biological processes, as well as advanced oxidation processes (AOPs), each with advantages and limitations. While AOPs are effective, they are often costly and may produce harmful by-products.

In recent years, green technologies such as adsorbents and photocatalysis have gained increasing attention. Unlike adsorption, which merely transfers pollutants between phases, the heterogeneous photocatalysis enables the complete mineralization of contaminants without generating toxic intermediates [5,6,7]. It further offers catalyst reusability, high stability, reduced operating costs, minimal solid waste and low energy consumption, making it both environmentally and economically sustainable [8]. Various semiconductors, including TiO_2_ [9], ZnO [10], hematite and iron oxides [11], g-C_3_N_4_ [12], and tungstate or molybdate-based materials [13,14,15,16,17], have been investigated for pollutant degradation. TiO_2_ and ZnO remain the most studied due to their stability and low cost, but their activity is limited to the UV range unless doped.

Recent advances in photocatalysis for textile and wastewater treatment have therefore focused on heterostructured systems to improve charge separation and extend visible-light response. Examples include S-scheme heterojunctions for antibiotic degradation [18], core–shell CdS@ZnInS_4_ nanocomposites for dye reduction and stabilization against photocorrosion [19], Mn-doped Cd_1−x_S nanorods with optimized band gap engineering [20] and MIL-101(Fe)/Bi_2_MoO_6_ heterostructures for tetracycline removal [21].

Iron tungstate (FeWO_4_), also known as ferberite, is a wolframite-type ternary semiconductor [22,23,24,25,26,27] with intrinsic ferromagnetism, efficient visible light absorption, and promising photocatalytic activity [28]. Several studies report its use for degrading dyes such as methylene blue (MB), methyl orange, and rhodamine B under visible or simulated solar irradiation [22,23,24,25,29]. However, pristine FeWO_4_ usually shows limited catalytic activity, prompting research into dopants or heterostructures to boost performance. Examples include halogen ion doping, coupling with graphene oxide (GO) or forming heterostructures such as FeWO_4_/g-C_3_N_4_ or FeWO_4_/BiOCl to facilitate electron transfer and reduce electron–hole pair recombination, which significantly accelerate dye degradation—reported rate being several times faster than those obtained with bare FeWO_4_ [30,31,32].

These approaches confirm the interest in FeWO_4_ but rely on complex and costly modifications. In contrast, FeWO_4_ itself remains underexplored, especially regarding its growth mechanism and the role of synthesis parameters in controlling morphology and photocatalytic activity [22,25,33,34,35]. Previous hydrothermal studies [22,23,24,25,29,36,37] have only rarely addressed formation pathways, with notable exceptions being Fang et al. [35], who examined pH-dependent synthesis using iron ammonium sulfate, and Zhang et al. [33], who proposed a mechanism involving Fe–EDTA complexes.

Building on this background, the present work highlights that even pristine FeWO_4_, obtained from common precursors under mild hydrothermal conditions, can achieve rapid and efficient MB degradation under solar irradiation. A key originality lies in showing that morphological control alone, governed solely by the physical form of the FeSO_4_·7H_2_O precursor (solid vs. dilute), is sufficient to tune photocatalytic performance. This simple adjustment drives a dual growth mechanism, leading to two distinct morphologies (nanoplatelets and self-organized). Despite their relatively low surface area, both morphologies achieved near-complete MB degradation within minutes, with only low doses of H_2_O_2_. This distinguishes our work from previous reports where FeWO_4_ performance improvements required doping, heterostructures, or supports.

Here, a combination of structural, optical, electrochemical, kinetic, and radical analyses is employed to elucidate the growth mechanism of FeWO_4_ and clarify its photocatalytic activity. We examined the influence of pH and varied the oxidant concentration. Radical scavenging tests and electrochemical characterization (photocurrent response and Mott–Schottky analysis) were combined with band alignment to propose a consistent photo-Fenton mechanism. This integrated approach not only provides new insights into ferberite growth and activity, but also highlights its potential as an efficient, cost-effective, and scalable photocatalyst for textile wastewater treatment.

## 2. Results and Discussion

### 2.1. Structural and Chemical Characterization of Ferberite

**Figure 1:**
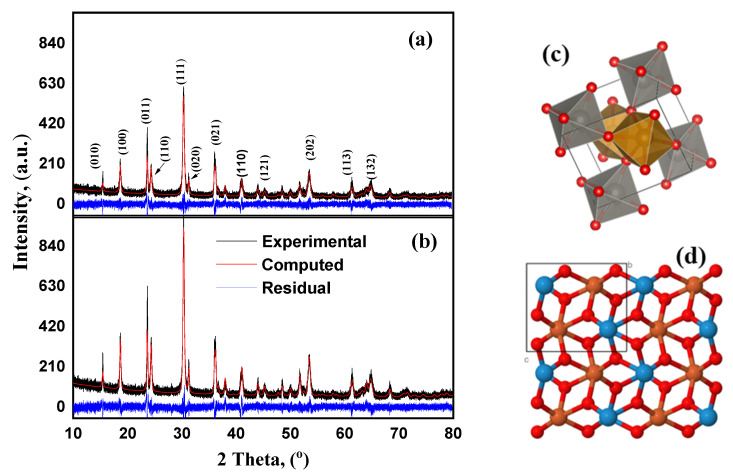
X-ray diffraction patterns of synthesized ferberite: (**a**) self-organized morphology and (**b**) nanoplatelets. (**c**) Coordination polyhedral (WO_6_ and FeO_6 o_ctahedral units) and (**d**) three-dimensional representations of the monoclinic unit cell of FeWO_4_.

Single-crystal ferberite powders were synthesized by introducing iron (II) sulfate heptahydrate either in solid form or as an aqueous solution, resulting in two distinct morphologies: self-organized microstructures composed of platelets, and isolated nanoplatelets. Figure 1 displays the X-ray diffraction (XRD) patterns and corresponding refinement results for the crystallized samples self-organized (a) and platelets (b). It is important to note that, regardless of the resulting morphology, the formation of the ferberite phase requires the careful optimization of synthesis parameters, including the type and concentration of precursors and solvents, reaction medium pH, and hydrothermal temperature. The synthesis conditions reported herein were specifically optimized to obtain phase-pure ferberite, free from secondary phases or impurities.

According to the XRD patterns shown in Figure 1, both samples exhibit well-crystallized structures with no detectable secondary phases, confirming the formation of a pure monoclinic phase with space group P2/c:1, consistent with the ICSD 15193 reference pattern. The diffraction profiles both in terms of peak intensity and width vary depending on the synthesis conditions, particularly whether iron (II) sulfate was introduced in solid form or as a solution. Differences in peak broadening between the samples reflect variations in crystallite size, as summarized in Table 1. Notably, the self-organized morphology displays broader and less intense peaks compared to the platelets, suggesting either lower crystallinity, smaller crystallite size, or a preferred crystallographic orientation associated with the anisotropic growth of platelets.

The structural and angular parameters, along with the crystallite size, were determined via Rietveld refinement using the MAUD software (version 2.064), based on the identified monoclinic space group P2/c:1. In Figure 1, the solid black line represents the experimental XRD data, the red line corresponds to the calculated pattern obtained from the Rietveld refinement, and the blue line indicates the difference (residual) between the experimental and calculated intensities. The refined crystallographic parameters, lattice constants, and reliability factors are summarized in Table 1.

The analysis of lattice parameters, crystallite size, and theoretical density presented in Table 1 indicates that the results obtained from the Rietveld refinement are consistent with values reported in the literature [28,37,38]. The quality of the refinement is supported by the reliability indicators: Rwp, Rexp, and the goodness of fit (Rwp/Rexp). Based on these values, the Rietveld refinement can be considered statistically reliable.

The refined lattice parameters obtained from the Rietveld analysis were used in the VESTA software (Visualization for Electronic and Structural Analysis, Version 3.90.0a) to construct the geometric model and evaluate interatomic distances and bond angles. The crystal structure of monoclinic FeWO_4_ is illustrated in Figure 1b. It features a multilayered arrangement composed of alternating polyhedral units and linear atomic chains involving Fe, W, and O atoms. In this representation, both W and Fe atoms are octahedrally coordinated by oxygen, forming WO_6_ and FeO_6_ structural units. The coordination parameters derived from the Rietveld refinement enabled the determination of bond lengths, with W–O distances ranging from 1.883 to 2.109 Å and Fe–O distances from 2.003 to 2.170 Å. The associated bond angles [O–W(Fe)–O] vary between 40° and 166°, indicating significant distortions in the polyhedral environment. These distortions are characterized by a stronger contraction of the WO_6_ octahedra and a relative elongation of the FeO_6_ units.

The synthesized structures exhibited good structural stability and retained their morphology after ultrasonic dispersion, suggesting strong chemical bonding within the polyhedral framework. TEM-EDX analyses were conducted to assess the local chemical composition and atomic distribution of Fe and W across different microcrystalline regions. Regardless of morphology self-organized or nanoplatelets, the EDX results confirmed a stoichiometry consistent with the expected Fe:W atomic ratio in FeWO_4_. Specifically, the self-organized samples contained 47% Fe and 53% W, while the platelet-shaped samples exhibited 48% Fe and 52% W. Notably, achieving the correct stoichiometry for phase-pure ferberite using the synthesis method reported herein required an initial iron concentration twice that of tungsten and oxalic acid.

Ferberite has also been synthesized via hydrothermal methods using different precursors, such as ferrous ammonium sulfate [(NH_4_)_2_Fe(SO_4_)_2_·6H_2_O] and sodium tungstate [Na_2_WO_4_·2H_2_O], followed by hydrothermal treatment at 190 °C for 24 h across a wide pH range (1–12) [23]. However, ferberite was not formed under all conditions: at pH 1, WO_3_ was obtained, while at pH 12, Fe_2_O_3_ was the predominant phase. In contrast, in our study, the pure ferberite phase was successfully obtained at pH 1. This discrepancy could be primarily attributed to the nature of the iron precursor, as the hydrothermal temperatures used in both cases were comparable (approximately 200 °C). Kovács et al. [39] also reported the critical influence of the iron precursor on the hydrothermal synthesis of ferberite, demonstrating that even under otherwise identical conditions, changing the iron source alone could inhibit the formation of the FeWO_4_ phase.

### 2.2. Morphological Characterization of Ferberite

**Figure 2:**
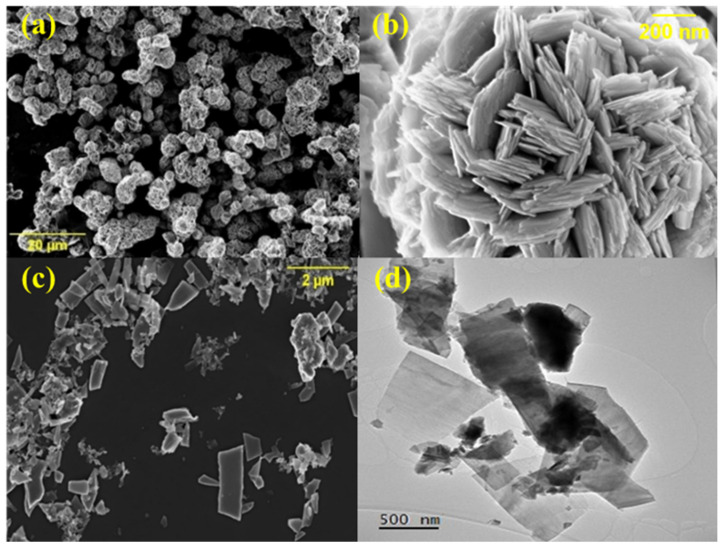
SEM/TEM of ferberite (**a**,**b**) self-organized and (**c**,**d**) platelets.

Figure 2 presents the morphological characterization of the ferberite samples, which exhibit self-organized desert rose-like structures and isolated nanoplatelet morphologies. These features were analyzed by scanning electron microscopy (SEM, images (a)–(c) and transmission electron microscopy (TEM, image (d)). The electron diffraction patterns of the self-organized/platelet morphologies are shown in Figure S1 (TEM, Supplementary Material). The SEM images were acquired at different magnifications to highlight structural details: 1.2 k× for image (a), and 70 k× for images (b) and (c).

As observed in the SEM images, the self-organized ferberite sample primarily consists of microstructures resembling desert roses, characterized by radially arranged platelets forming rosette-like architectures (Figure 2a,b). These results are consistent with those reported by Kovács et al. [39]. In contrast, the second sample, obtained using diluted iron(II) sulfate heptahydrate, primarily exhibits irregular rectangular and square-shaped platelets with varying sizes (Figure 2c,d). While the self-organized morphology shows a more uniform size distribution, the individual platelets in the second sample are considerably smaller, despite their irregular shape. The morphological contrast between Figure 2a,c clearly highlights the influence of the physical form of the iron precursor solid or dissolved on the resulting ferberite morphology, even in the presence of oxalic acid. This simple variation in precursor introduction leads to a transition from well-organized rosette-like assemblies to disordered and isolated nanoplatelets. Selected-area electron diffraction (SAED) patterns confirm that the plates for both samples are single crystals with a (100) zone axis aligned along the *a*-axis. This indicates preferential two-dimensional growth along the *b* and *c* directions, with growth suppression along the large surface area. This anisotropic growth behavior is primarily driven by the presence of oxalic acid and further modulated by whether the iron precursor is added in diluted solution or solid form.

### 2.3. Growth Mechanism of Ferberite Catalyst

The formation mechanism of ferberite was investigated by analyzing samples collected at different stages of growth using microscopy and infrared spectroscopy. The hydrothermal synthesis produced two distinct morphologies: isolated nanoplatelets and self-organized aggregates resembling desert rose-like architectures. Experimental evidence suggests that the physical form in which iron (II) sulfate heptahydrate is introduced either as a solid or in solution plays a key role in directing the morphological evolution toward either individual platelets or their self-organized into rosette-like structures. Overall, both morphologies appear to originate from the initial formation of single-crystalline platelets.

#### 2.3.1. Sodium Tungstate Dihydrate Dissolution and Role of Oxalic Acid

**Figure 3:**
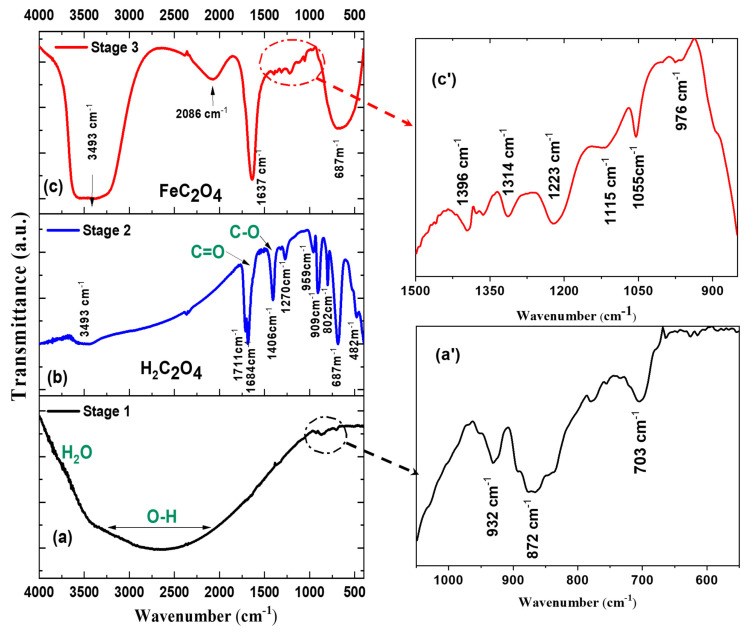
FTIR spectra corresponding to (**a**) stage 1 (acidification of a sodium tungstate solution to pH 1 with 3 M HCl), (**a’**) zoom section of spectra (**a**,**b**) stage 2 (addition of oxalic acid), (**c**) stage 3 (addition of iron sulfate heptahydrate) of the synthesis methodology and (**c’**) zoom section of spectra (**c**).

Initially, sodium tungstate dihydrate was dissolved as described in Equation (1), resulting in a clear solution. Subsequent acidification with HCl to pH 1 leads to the protonation of tungstate ions (Equation (2)). At this first stage, the solution undergoes a visible color change from colorless to pale green, indicating the formation of hydrated tungstic acid species (H_2_WO_4_·nH_2_O), as represented in Equation (3). In the second stage, the WO_5_(H_2_O) units become further hydrated and undergo dimerization through oxygen bridges, forming crystalline WO_3_·2H_2_O, in which tungsten centers are octahedrally coordinated. The WO_3_·2H_2_O structure consists of layers of corner-sharing WO_5_(H_2_O) octahedra separated by interlayer water molecules. Each WO_5_(H_2_O) octahedron contains four W–O single bonds, one terminal W=O double bond, and one W–OH_2_ bond, where the tungsten center is coordinated in relation to a water molecule. The interlayer water molecules form hydrogen bonds with the WO_5_(H_2_O) units, contributing to the layered architecture of the WO_3_·2H_2_O phase. Upon the removal of the interlayer water, orthorhombic H_2_WO_4_ is formed, as previously reported in the literature [33]. This transformation step is further supported by the infrared spectroscopy data presented in Figure 3a,a’. The vibrational spectrum displays a broad absorption band in the 3100–3550 cm^−1^ region, the characteristics of O–H stretching vibrations associated with the hydrated phase. Additionally, a distinct band corresponding to the W=O stretching mode is observed at 872 cm^−1^, while O–W–O bending vibrations appear at 932 cm^−1^. These features confirm the presence of tungstate-based hydrated species in the sample.

(1) $$2{Na}_{2}WO_{4}\cdot 2H_{2}O\to 4{Na}^{+}+2WO_{4}^{2-}+\left(n+2\right)H_{2}O$$

(2) $$2{Na}^{+}+WO_{4}^{2-}+2H^{+}+2{Cl}^{-}\to 2NaCl+WO_{5}(OH_{2})$$

(3) $$2{Na}^{+}+WO_{4}^{2-}+2HCl+nH_{2}O\to 2{Na}^{+}+2{Cl}^{-}+H_{2}WO_{4}\cdot 2H_{2}O$$

Upon the addition of oxalic acid (H_2_C_2_O_4_), the solution becomes colorless. This change reflects the strong interaction between oxalate ions (C_2_O_4_^2−^) and the interlayer water molecules in WO_3_·2H_2_O. The oxalate species disrupt the hydrogen bonding network between the water molecules and the WO_5_(H_2_O) octahedra, thereby accelerating the dehydration of WO_3_·2H_2_O into the intermediate WO_3_·H_2_O phase. A similar oxalate-induced dehydration mechanism has been reported by Li et al. [40]. This transformation is further supported by FTIR analysis (Figure 3), which reveals characteristic vibrational bands. These include a broad absorption band in the 2100–3550 cm^−1^ range, attributed to O–H stretching vibrations from structural water; a ν(W=O) stretching band at 872 cm^−1^; and O–W–O bending modes at 689 and 800 cm^−1^. Additional bands corresponding to oxalic acid are observed at 909, 1270, 1406, 1684, and 1711 cm^−1^, associated with ν(C–O), ν(C=O), and ν(O–C–O) vibrational modes [34].

Oxalic acid, which contains two carboxyl groups, is known to form bidentate chelates with tungsten species through O–W–O bridges, thereby stabilizing acidic tungstate solutions and suppressing premature polycondensation [41,42]. This chelation effect promotes the selective stabilization of specific crystal growth planes, thereby influencing both the nucleation and anisotropic growth of WO_3_·nH_2_O during the early stages of the hydrothermal process. Notably, Fang et al. [43] demonstrated that oxalic acid dihydrate can act as both a reactant and a lamellar template in the mechanochemical synthesis of WO_3_·2H_2_O ultrathin nanosheets. Although their solvent-free approach differs from the present hydrothermal method, their findings reinforce the critical role of oxalate species in directing anisotropic growth and controlling the morphology of tungsten oxide hydrates. A greater detailed description is provided in Section 2.3 for stage 3.

#### 2.3.2. Nucleation and Growth Mechanism: Insights from TEM and FTIR Analyses

**Figure 4:**
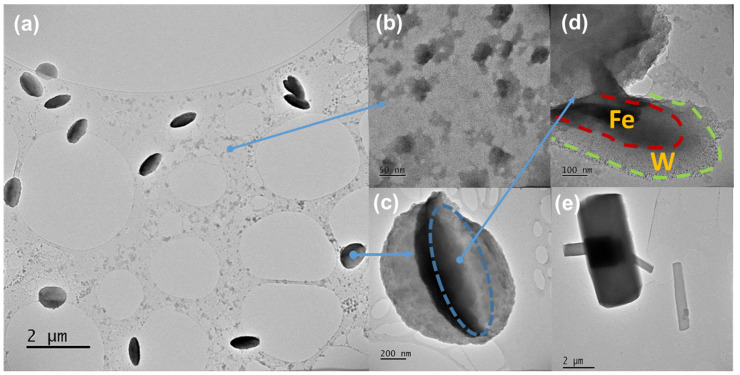
TEM images at different stages of ferberite growth (**a**) after mixing the precursors corresponding to step 3. (**b**) An amorphous zone of the sample. (**c**) Disk-forming stage and (**d**) growth form and formation of platelets. (**e**) Goethite-like platelets formation only with iron precursor.

Transmission electron microscopy (TEM) analyses of the morphology and composition of aliquots collected before the hydrothermal treatment revealed clear morphological differences, as shown in Figure 4a,d. Additional analyses were carried out to observe the behavior of iron sulfate alone under the same growth conditions. This resulted in the formation of iron or iron hydroxide platelets, as reported in Figure 4e. Some goethite-like platelets were also identified (Figure 4e). TEM analysis conducted during the early stages of synthesis (Figure 4) revealed that the addition of iron sulfate heptahydrate induced the formation of fusiform amorphous aggregates, along with a film-like amorphous phase (as verified by the absence of the electronic diffraction pattern of these observed areas).

Energy-dispersive X-ray spectroscopy (EDS) confirmed that the amorphous film (Figure 4a) contained approximately 50% W, 40% Fe, and 10% S (oxygen not quantified). The aggregates (Figure 4c,d) exhibited an Fe- and S-rich core surrounded by a tungsten-rich shell, supporting the hypothesis of core–shell-type nucleation followed by interdiffusion processes.

As the reaction proceeded, these mixed amorphous precursors progressively reorganized and crystallized into platelet-like structures. TEM images recorded at later stages (not presented) show that the interdiffusion and dehydration processes led to the development of well-defined lamellar morphologies, consistent with the platelet architecture observed in the final product. This sequence of transformations highlights the critical role of the initial heterogeneous nucleation in promoting the formation of crystalline nanoplatelets from amorphous intermediates. FTIR spectra collected during this stage (Stage 1, Figure 3a,a’ displayed broad bands around 2100–3500 cm^−1^, corresponding to O–H stretching vibrations from structural water. Vibrations associated with C–O and C=O bonds of oxalic acid were detected near 1300 and 1650 cm^−1^, further confirming the presence of oxalate complexes species.

#### 2.3.3. Proposed Dual-Pathway Growth Mechanism

Both observed morphologies individual nanoplatelets and self-organized rosette-like architectures are monoclinic, single-crystalline FeWO_4_, as confirmed by XRD, TEM, and EDS analyses. The main difference lies in how iron sulfate heptahydrate is introduced into the reaction medium, which decisively influences nucleation and aggregation phenomena. This observation suggests that the two morphologies originate from a common set of chemical transformations (Equations (4)–(6)), diverging only at the stage of iron incorporation and particle assembly.

(4) $$2C_{2}O_{4}^{2-}+WO_{4}^{2-}+6H^{+}\to C_{4}H_{4}O_{4}WO_{4}\cdot 2H_{2}O$$

(5) $$C_{4}H_{4}O_{4}WO_{4}\cdot 2H_{2}O+Fe{\left(OH\right)}_{2}\to {Fe}^{2+}+WO_{4}^{2-}+2H^{+}+C_{2}O_{4}^{2-}$$

(6) $${Fe}^{2+}+WO_{4}^{2-}+2H^{+}+C_{2}O_{4}^{2-}\to 2H^{+}+C_{2}O_{4}^{2-}+FeWO_{4}$$

**Scheme 1:**
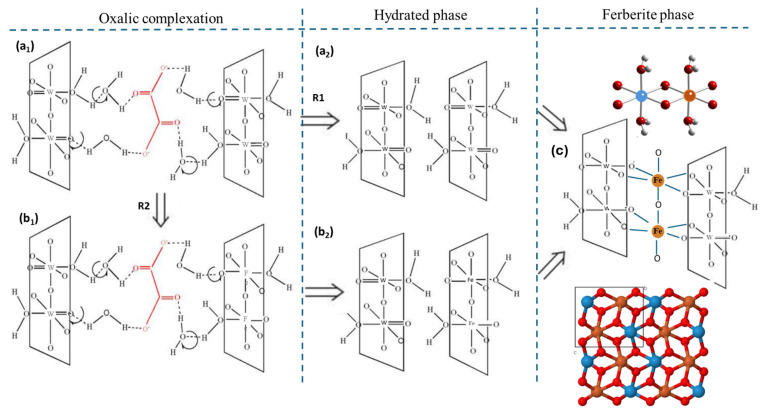
Growth mechanism of ferberite. (**a_1_**) Tungstate oxalate. (**a_2_**) Tungstate hydrate. (**b_1_**) Iron–tungsten oxalic complexation. (**b_2_**) Iron–tungsten hydrate and (**c**) final ferberite formation step. Reproduced with permission from Ref. [33]. Copyright Elsevier. 2011.

Scheme 1 illustrates this dual-pathway mechanism, consisting of two parallel reaction routes. The first step corresponds to the tungsten oxalate complexation (a1) and (a2), as reported by Li et al. [40]. We then suggested the following steps based on our observations.

**Pathway A1 → A2 (platelet growth):**

In this route, iron sulfate heptahydrate was introduced in a dilute aqueous form. Sodium tungstate reacts with oxalic acid under acidic conditions, leading first to the formation of a tungsten–oxalate complex. Subsequent hydrothermal treatment and progressive dehydration result in the transformation of this precursor into hydrated WO_3_ platelets. Iron ions interact via hydrogen bonds with structural water. This promotes the nucleation and growth of FeWO_4_ nanocrystals while preserving platelet morphology.

In this scenario, oxalic acid plays a critical role in promoting 2D growth by stabilizing specific faces of the WO_3_ nuclei through hydrogen bonding. The carboxyl groups on oxalate interact with tungsten precursors, effectively isolating the nuclei and inhibiting their aggregation, as previously reported [41,44,45].

**Pathway A1 → B1 (self-organized):**

When iron sulfate heptahydrate is added in solid form (stage 3), a localized excess of iron and sulfate ions is generated in the immediate vicinity of tungstate–oxalate complexes. TEM images (Figure 4c,d) show that iron sulfate acts locally as a nucleation center, being rapidly coated by a shell of tungsten oxalate and hydrated WO_3_. This heterogeneous nucleation yields core–shell aggregates enriched in Fe and S (as confirmed by EDS). Under these conditions, the combination of strong hydrogen bonding between oxalate ions and structural water, and high local supersaturation of Fe^2+^, promotes the aggregation of nascent platelets into interconnected disks. As hydrothermal ripening proceeds, these assemblies evolve into rosette-like superstructures. FTIR spectra recorded at this stage exhibit characteristic O–H and C–O vibrations (bands at ~1300 cm^−1^ and ~1650 cm^−1^, as shown in Figure 3c,c’), further confirming the presence of oxalate ligands mediating the assembly process.

#### 2.3.4. Influence of Iron Precursor Physical Form on Growth Mechanism and Morphology

The final morphology of the ferberite particles was found to be strongly dependent on the physical form in which iron sulfate heptahydrate was introduced into the oxalate–tungstate reaction medium. When added as a diluted aqueous solution, homogeneous nucleation and controlled interdiffusion promoted the growth of isolated, well-dispersed nanoplatelets. Under these conditions, oxalic acid acted as a stabilizing ligand, selectively adsorbing onto specific crystal facets and inhibiting lateral aggregation through hydrogen bonding interactions. This pathway favored a controlled two-dimensional growth of lamellar structures, consistent with previous reports on oxalate-assisted morphological control in tungsten oxide nanostructures [40]. In contrast, the direct addition of solid iron sulfate heptahydrate created localized supersaturation and heterogeneous nucleation sites, leading to amorphous mixed aggregates with core–shell-like composition. Subsequent interdiffusion, dehydration, and recrystallization transformed these precursors into interconnected platelets that progressively self-organized into rosette-like hierarchical architectures. Oxalate ions further facilitated this organization by bridging adjacent nanoplatelets through hydrogen bonds with interlayer water.

Overall, the morphology emerged from the interplay between oxalic acid complexation and the nucleation environment dictated by the iron precursor form. This dual mechanism accounts for the transition from isolated nanoplatelets to hierarchical structures, as summarized in Scheme 1.

### 2.4. Textural Properties: Surface Area and Porosity

**Figure 5:**
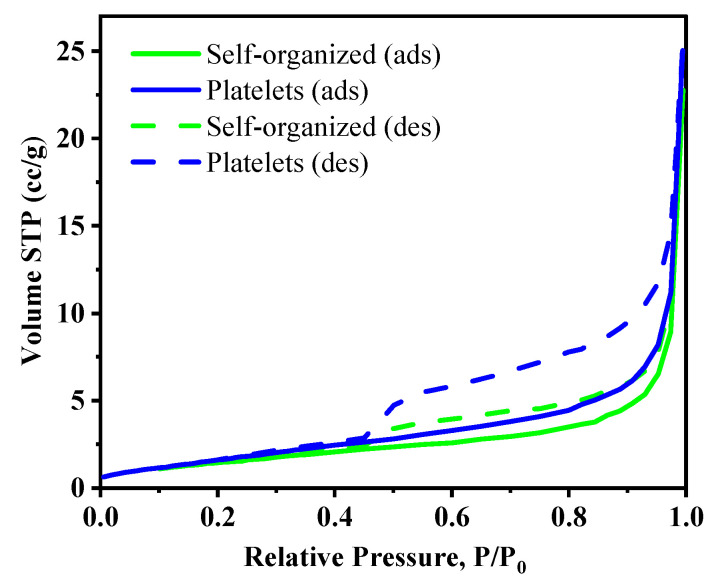
N_2_ adsorption/desorption isotherm of ferberite self-organized and platelets.

The specific surface area and porosity of the synthesized FeWO_4_ materials, with distinct self-organized and platelet morphologies, were investigated using nitrogen (N_2_) adsorption–desorption process. Figure 5 shows the N_2_ adsorption–desorption isotherms of each morphology. The FeWO_4_ self-organized and platelet morphologies exhibited Type IV isotherms with an H3-type hysteresis loop, in accordance with the IUPAC classification [46].

The specific surface area (S_BET_) was calculated using the Brunauer–Emmett–Teller (BET) method [47]. The average pore diameter and the total pore volume were determined using the Barrett–Joyner–Halenda (BJH) method from the desorption branch of the isotherms [48]. The FeWO_4_ with platelet morphology exhibited a specific surface area of 12.7 m^2^g^−1^, almost double that of self-organized FeWO_4_ (6.80 m^2^g^−1^). Similarly, the platelet morphology exhibited a total pore volume that was 17% larger (0.041 cm^3^g^−1^) than that of the self-organized morphology (0.035 cm^3^g^−1^). However, the average pore diameters for both samples were nearly similar, around 19 Å.

These textural characteristics, particularly the specific surface area and pore volume, are known to be influenced by the synthesis method and its parameters [49]. The larger surface area and pore volume observed for the platelet FeWO_4_ are generally considered advantageous for photocatalysis, as they can provide more active sites for reactant adsorption and facilitate the diffusion of reactants and products [22,50].

### 2.5. Optical Properties—UV-Vis Diffuse Reflectance

The electronic band structure of ferberite arises from the overlap of atomic orbitals of its constituents. As reported in the literature, Fe 3d orbitals dominate the top of the valence band, while the bottom of the conduction band is primarily composed of hybridized W5d and O2p states [51]. Structural variations, including those induced by synthesis conditions and morphology, can reduce the band gap energy, typically attributed to an enhanced hybridization between Fe^2+^ 3d and O 2p orbitals.

**Figure 6:**
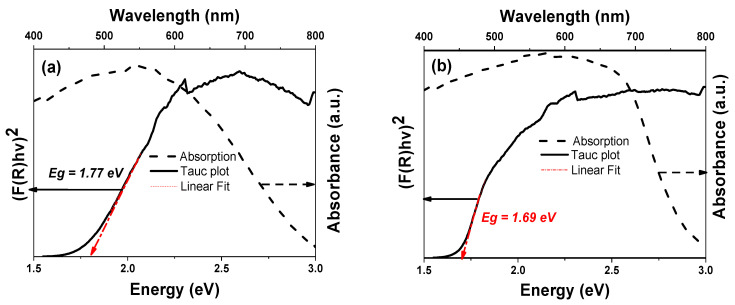
Tauc plots and absorption spectra of FeWO_4_: (**a**) nanoplatelets and (**b**) self-organized.

To assess the light-harvesting capabilities of FeWO_4_ with both distinct morphologies (self-organized vs. isolated platelets), UV–visible diffuse reflectance spectra (DRS) were recorded (Figure 6). Band gap energies (Eg) were estimated using the Tauc relation Equation (S1) (in the Supplementary Materials).

The nature of the band gap in FeWO_4_ remains debated. Some band structure studies predict a direct transition [52,53] while others support an indirect one [23,35]. These discrepancies are often linked to differences in synthesis method, grain size, morphology, and microstructuring, as confirmed in DFT-based studies. For self-organized FeWO_4_, the band gap was found to be 1.62 eV, assuming a direct transition, and 1.69 eV, assuming an indirect transition. In contrast, the platelet morphology exhibited a slightly lower value of 1.47 eV for the direct transition, and 1.77 eV for the indirect one. These values fall within the broad range of band gaps reported for FeWO_4_, which vary depending on synthesis methods, e.g., 1.7 eV (microemulsion) [24], 2.96 eV (precipitation) [25], 2.1 eV (solvothermal), and up to 3.17 eV (sol–gel) [26]. In hydrothermal conditions, reported band gaps range from 1.53 eV to 2.4 eV [22], with intermediate values such as 1.98 eV also reported for amine-assisted synthesis [54]. Overall, the literature reports indicate that the E_g_ of FeWO_4_ varies significantly depending on the assumed transition type: 1.6–2.3 eV for direct transitions and 1.3–2.0 eV for indirect ones, reflecting the influence of synthesis routes and microstructural factors.

The results in Figure 6 show the Tauc graph of FeWO_4_ with the band gap of 1.69 eV and 1.77 eV for self-organized and platelets. Based on the literature and our experimental spectra (Figure 6a), which show a single dominant linear region, we consider a direct band gap model to be most appropriate for our hydrothermally synthesized ferberite [24,25,29,55]. This assumption will be used in subsequent energy band diagram discussions.

### 2.6. Photocatalytic Activity of Ferberite with Self-Organized and Platelet Morphologies

#### 2.6.1. Adsorption Studies

Before testing photocatalytic performance, the interaction between methylene blue (MB) molecules and the catalyst surface in the absence of light was investigated to distinguish adsorption effects from true photocatalytic degradation. Adsorption–desorption equilibrium was assessed through dark experiments. Figure S2 presents the evolution of the UV-Vis absorption spectra of MB over time in the Supplementary Materials. Regarding platelet morphology, equilibrium was reached within 10 min, with no significant variation in MB observed up to 60 min. During this period, approximately 30% of the MB was removed via adsorption (Figure S2a, in the Supplementary Materials). Based on these observations, a 20 min dark stirring step was systematically applied before initiating all photocatalytic degradation tests to ensure equilibrium. It is noteworthy, however, that under irradiation, the photodegradation of MB by both ferberite morphologies-platelets or self-organized structures, remained limited. These results are detailed in the next section.

#### 2.6.2. Influence of pH and H_2_O_2_ on MB Degradation

**Figure 7:**
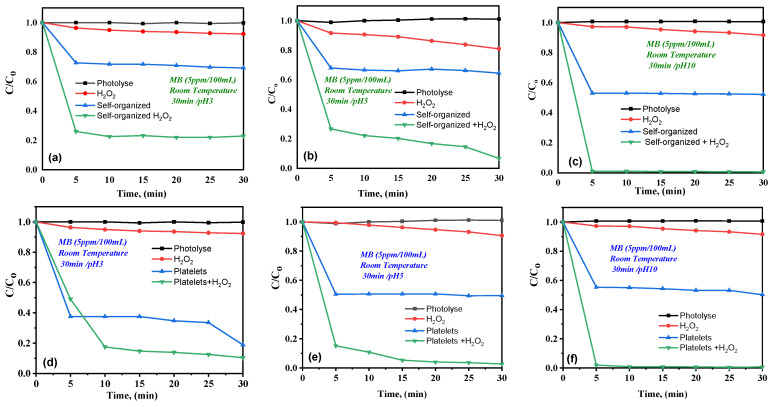
Catalytic activities of FeWO_4_ under various conditions for MB dye photodegradation: photolysis without catalyst, with H_2_O_2_ addition, with ferberite and ferberite/H_2_O_2_ system. Self-organized (**a**–**c**) and platelets (**d**–**f**), at pH 3, 5 and 10, respectively.

The photocatalytic degradation of MB activated by FeWO_4_ with both self-organized and platelet morphologies was investigated under visible light, with and without hydrogen peroxide (H_2_O_2_), and at varying initial pH values (3, 5 and 10). Figure 7 shows the evolution of MB relative concentration (C/C_0_) over time under various conditions: photolysis (MB alone) without catalyst, FeWO_4_ alone, and the FeWO_4_ combined with H_2_O_2_ (photo-Fenton system) for both morphologies (Figure 7a–f. It should also be noted that, in the absence of light, the H_2_O_2_-assisted catalysts show no additional catalytic activity compared with the adsorption stage.

- Blank tests under visible light (photolysis, H_2_O_2_ alone, FeWO_4_ alone)

The direct photolysis of MB or degradation using H_2_O_2_ alone led to negligible removal (ca. 9% after 30 min with H_2_O_2_ alone). In contrast, FeWO_4_ alone promoted partial degradation: self-organized FeWO_4_ achieved 25–45% degradation, while platelet-shaped FeWO_4_ reached 45–65%, depending on pH. In both cases, photocatalytic activity increased with rising pH, suggesting more efficient degradation pathways in basic media. Nonetheless, the overall performance of FeWO_4_ alone remained moderate, likely limited by low surface area, electron–hole pair recombination, and insufficient ROS radical production.

b. Effect of H_2_O_2_ addition (photo-Fenton conditions)

A marked improvement was observed upon H_2_O_2_ addition. Under photo-Fenton conditions, MB degradation increased, reaching 75% to 100% within 15 min, as the pH rose from acidic to basic, regardless of morphology. This enhancement reflects the strong synergistic effect between FeWO_4_ and H_2_O_2_ (Figure 7), especially in acidic or neutral media. This synergy arises from the dual action within the photo-Fenton mechanism: photogenerated electrons in the FeWO_4_ conduction band that can react with H_2_O_2_ to form hydroxyl groups (Equations (7) and (8), respectively).

(7) $$FeWO_{4}+hv\to e_{CB}^{-}+h_{VB}^{+}$$

(8) $$e_{CB}^{-}+H_{2}O_{2}\to {OH}^{*}+{OH}^{-}$$

In parallel, Fe^2+^/Fe^3+^ surface sites catalyze the classical Fenton reactions (Fe^2+^ + H_2_O_2_ → Fe^3+^ + *OH + OH^−^), further boosting the generation of highly oxidizing ${OH}^{*}$ radicals.

c. pH influence and surface charge

**Figure 8:**
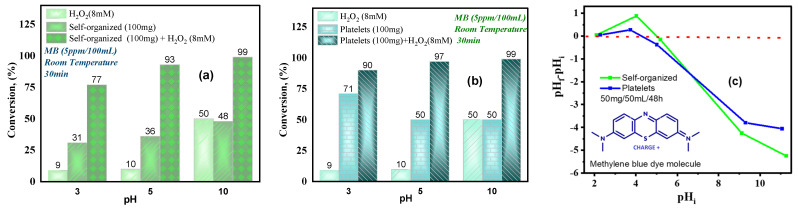
Effect of pH on photodegradation of MB with and without H_2_O_2_. (**a**) self-organized, (**b**) platelets, and (**c**) pH_p*zc*_ of self-organized and platelets with MB molecule.

Solution pH also plays a crucial role in the catalytic performance of ferberite. An overall increase in MB degradation was consistently observed as the pH increased from acidic to basic conditions. This effect was particularly pronounced for the self-organized FeWO_4_. This trend can be attributed to pH-dependent electrostatic interactions between the catalyst surface and the cationic dye MB, as revealed by the catalyst’s point of zero charge (pH_pzc_) determined for both morphologies. The pH_pzc_ of the ferberite materials was determined to be approximately 4.8 for the self-organized structures and 4.5 for the platelets (Figure 8c). At pH value above pH_pzc_, the catalyst surface becomes negatively charged, thereby promoting electrostatic attraction with the cationic MB molecules. This enhances dye adsorption and subsequent degradation.

d. Morphology effect

The comparison of the two morphologies (Figure 8) shows that platelet-shaped ferberite generally outperformed the self-organized form, particularly in the absence of H_2_O_2_. For example, at pH 3, the platelets achieved ca. 40% higher degradation than their self-organized counterpart. However, under neutral or alkaline conditions, both morphologies exhibited similar efficiencies. Under photo-Fenton conditions, degradation increased to 77% and 90% for self-organized and platelet morphologies, respectively, at pH 3, and nearly 100% at higher pH, regardless of the morphology. This enhanced photodegradation is attributed to the low band gap values of both samples (see Figure 6), which facilitate visible-light absorption. In addition, the superior performance of the platelet’s morphology can be linked to its favorable textural properties, namely its higher specific surface area and greater pore volume, which promote reactant adsorption and light harvesting.

As mentioned above, morphology has a direct impact on catalytic performance. Compared to the self-organized structure, the platelet morphology features larger crystallites (87 nm) and higher crystallinity. This is confirmed by the narrower diffraction peaks in the XRD pattern (Figure 1b), contrasting with the smaller crystallites (68 nm) found in the self-organized morphology. Consequently, platelets have a larger specific surface area, creating a greater number of active surface sites and a larger light absorption cross-section than self-assembled samples. Additionally, the larger surface area and pore volume of the platelets promote better adsorption of methylene blue (MB), improving the diffusion of H_2_O_2_ and O_2_. This results in the greater exposure of active sites, and the localized generation of OH^•^ radicals. Furthermore, the platelet morphology has a more negative conduction band potential of –0.05 V (as detailed in Section 2.7). This property, combined with a higher photocurrent, ensures greater availability of charge carriers, which effectively promotes the reduction of H_2_O_2_ to OH^•^ radicals. This explains the superior degradation activity observed under acidic conditions, even when the catalyst surface is positively charged.

e. Influence of H_2_O_2_ concentration

**Figure 9:**
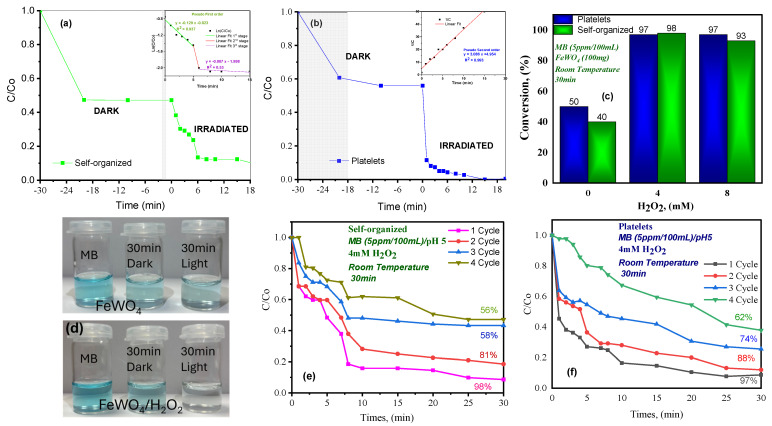
Photocatalytic degradation of MB at pH 5 with 4 mM H_2_O_2_: (**a**) self-organized microstructures, and (**b**) platelet morphology. Inserts: corresponding kinetic model fits, respectively, for self-organized (insert (**a**)) and platelet morphology (insert (**b**)). (**c**) Effect of H_2_O_2_ concentration on MB degradation. (**d**) Photograph color change in MB solution, after adsorption–desorption equilibrium and after 30 min of irradiation recyclability tests of (**e**) self-organized and (**f**) platelets samples.

Based on the analysis of the MB photodegradation results as a function of pH presented in Figure 8, a second study is presented to verify the influence of the H_2_O_2_ concentration present in the reaction medium (4 and 8 mM), as shown in Figure 9. Figure 9c shows the photodegradation rate (%) of the two different ferberite morphologies (platelets in blue and self-organized in green) as a function of the addition of H_2_O_2_ (0, 4 and 8 mM) in the photodegradation of MB. At low concentrations (4 mM), both perform well, achieving yields over 90% and indicating limited radical recombination. Both morphologies show almost similar degradation rates, with a slight drop of 2%, within the margin error. This shows that the 4mM concentration of H_2_O_2_ is close to the optimum concentration required for the photo-Fenton reaction. The result indicates that surface area (SA) is not the determining factor. In comparison to self-organized FeWO_4_, platelets possess nearly double the number of active sites based on SA.

f. Kinetic analysis

From a kinetic standpoint, the degradation mechanism of MB involves not only photogenerated charge carriers, but also the transport and diffusion of reactive oxygen species, such as hydroxyl radicals OH^•^ and hydrogen peroxide H_2_O_2_. Despite the rather low specific surface area of the FeWO_4_ sample, catalytic activity in the presence of H_2_O_2_ remained remarkably fast. Regardless of pH, MB degradation consistently exceeded 80% and reached 100% under acidic conditions. To obtain further insight into the reaction kinetics, additional experiments were conducted at a fixed H_2_O_2_ concentration of 4 mM. Figure 9d illustrates the color change between the initial MB solution, the solution after 30 min in the absence of irradiation (adsorption–desorption equilibrium), and the solution after 30 min of irradiation.

As shown in Figure 9a,b, the self-organized ferberite appeared to be slightly more efficient than its platelet-shaped counterpart, achieving a 10% higher uptake prior to illumination, but much slower degradation during the first 10 min of light exposure. A detailed examination of catalytic activity reveals different kinetic behaviors. For example, MB degradation by platelets occurs in one-step, whereas self-assembled samples require several steps. Moreover, kinetic constant representation revealed a difference in apparent reaction order between the two morphologies. The self-organized form followed pseudo-first-order kinetics, while the platelet morphology exhibited pseudo-second-order behavior. This is confirmed by the linear fits of the apparent rate constants for each material (platelets: k = 3.086 min^−1^; self-organized structures: k = 0.129 min^−1^) as reported in Figure 9a,b. The difference likely arises from the quantities and types of reactive oxygen species (ROS) involved, particularly between OH^•^ and H_2_O_2_. The structuring of ferberite influences the transport of H_2_O_2_ and intermediates (-OH). The presence of microporosity and extended platelet surfaces favors diffusion limited processes and accelerates surface reactions. Although these initial findings are promising, further investigation is needed to confirm the reaction orders and identify the dominant factors controlling the kinetics and reaction mechanism.

Increasing the catalyst dosage enhances the number of exposed active sites, improves MB adsorption, and promotes the generation of greater numbers of e^−^/h^+^ pairs. However, beyond an optimal threshold, the photocatalytic activity tends to plateau or even decrease due to the increased turbidity of the suspension (light scattering), particle shadowing, and agglomeration, which collectively reduce the effective active surface area. Therefore, in this study, we adopted a typical photocatalyst dosage of 1 g·L^−1^, in agreement with the literature [5]. Regarding the oxidizing agent, at low [H_2_O_2_] (4 mM) (Figure 9c), the system operates near its optimum condition, providing efficient OH^•^ generation without excess oxidant. Increasing [H_2_O_2_] to 8 mM leads to only a slight improvement in degradation, suggesting that the system approaches saturation in OH^•^ production.

g. Comparison with literature

In comparison, other studies in the literature using either pure or modified FeWO_4_ typically require longer reaction times, higher concentrations of H_2_O_2_, or additional conditions such as UV radiation, metal doping, or heterostructure formation. Qian et al. [22] employed FeWO_4_ nanorods synthesized via a simple hydrothermal method. This achieved approximately 90% degradation of MB in 60 min under visible light with 6 mM H_2_O_2_ at pH 3 demonstrating lower activity and a slower degradation rate compared to the present work. Similarly, Liu et al. [29] utilized a microemulsion hydrothermal route to synthesize FeWO_4_ nanorods, reaching only 75% degradation after 120 min, and notably without the use of H_2_O_2_.

Doped and hybrid systems have also been reported. In the first case, ferberite was doped with either nickel [24] or with iodide/graphene oxide (I^−^/GO) [30], achieving degradation efficiencies between 95% and 98%. However, these results were obtained under more restrictive conditions such as UV irradiation or extremely acidic media (pH 3), and required chemical modification steps that may compromise reproducibility and increase production costs. In the second case, the FeWO_4_/FeS_2_ hybrid system [36] achieved 97% degradation in 30 min, which is a value comparable to the present work. However, this involved the synthesis of a binary composite with more complex morphological control. The most efficient system reported among the reviewed studies was that of Sun et al. [49], which used sol–gel synthesized nanofibers combined with ultrasound irradiation, achieving 99% degradation in just 15 min. Nevertheless, the combined use of visible light and ultrasound significantly increases both the energy demand and operational complexity of the process. Table 2 provides a comparative overview of the materials and experimental conditions used in some of the works reported in the literature and in this study.

It is worth noting that, in addition to the conditions listed in Table 2 such as the type of material, irradiation source, pH, and reaction time, the photodegradation performance of the pollutant is also strongly influenced by other parameters of the photocatalytic system. These include the initial concentration of methylene blue (C_0_), the amount of catalyst used, the reaction temperature, the intensity and distance of the light source, and the geometry of the photoreactor.

h. Recyclability and stability

Figure 9e,f display the recycling performance of ferberite catalysts with platelets and self-organized morphologies. The results reveal that the platelets catalyst exhibits superior cyclic stability, retaining higher activity up to the third cycle. In contrast, the self-organized catalyst undergoes a more pronounced deactivation during the early cycles. The catalysts stability was also investigated by FTIR (Figure S3a) and XRD (Figure S3b) analyses after using samples for photodegradation testing. The IR absorption bands and Bragg diffraction positions remain the same as before use, showing that the catalysts remain stable without any structural modification.

To assess chemical stability and leaching of the catalyst, the reaction solution was analyzed by Inductively Coupled Plasma Optical Emission Spectrometry (ICP-OES) after the final degradation cycle. Measurements were taken for 100 g of catalyst dispersed in 100 mL of solution.

Concentrations of iron cations measured at pH 5 and 10 show a mean value of 14 and 95 µg/L, respectively, for platelets and self-assembled samples. These values remain relatively negligible, particularly for platelets. However, at pH 3, in an acidic medium, the concentrations were high, in excess of 500 µg/L, reflecting the instability of both catalysts in acidic media. The low concentrations of iron ions in the solution indicate that the leaching phenomenon through the cycles is negligible for platelets. The ability to maintain high efficiency over multiple cycles is due to the high stability of the catalyst and demonstrates the Ferberite efficiency for practical photodegradation applications.

i. Radical scavenging experiments

To identify the main reactive chemical species responsible for MB degradation, radical scavenging experiments were performed. Disodium ethylenediaminetetraacetic acid (EDTA), isopropyl alcohol (IPA) and benzoquinone (BQ) were used as specific quenchers for photogenerated holes (h^+^), hydroxyl radicals (OH^•^), and superoxide radicals ($O_{2}^{-•})$, respectively [59]. A significant reduction in degradation rate upon the addition of a given scavenger indicates the involvement of the corresponding species in the photodegradation mechanism.

**Figure 10:**
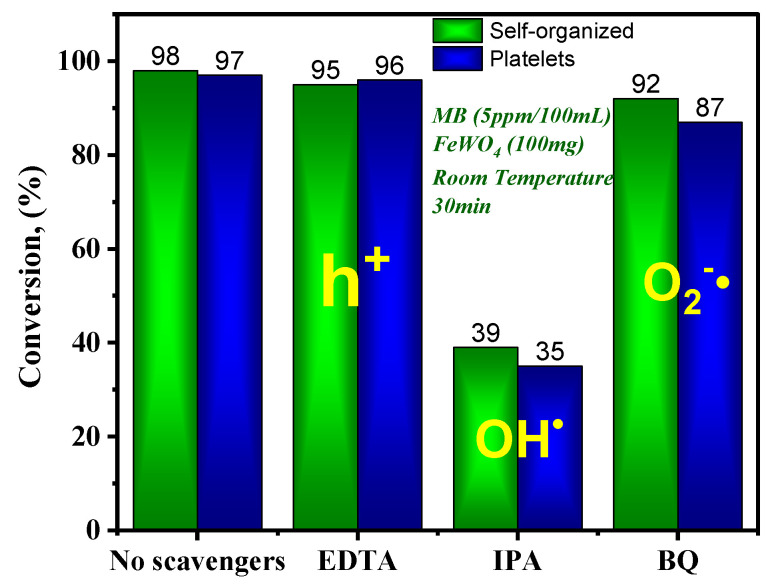
Radicals trapping test by ferberite (self-organized and platelet morphologies) (measurements after 30 min).

Figure 10 demonstrates first that there is no significant difference between the two morphologies (self-organized and nanoplatelets), then highlights the central role of OH^•^ in MB degradation mechanism under photo-Fenton conditions. The addition of IPA led to a substantial decrease in degradation activity, approximately 39% for the self-organized form and 35% for the platelet’s morphology indicating that hydroxyl radicals are the main reactive species with both catalysts. In contrast, the addition of EDTA and BQ resulted in only minor reductions (less than 10%), indicating that removing holes or superoxide radicals does not substantially affect photodegradation.

The involvement of OH^•^ radicals is consistent with classical Fenton-type reactions occurring at Fe^2+^/Fe^3+^ sites on the catalyst surface (Equations (9) and (10)):

(9) $${Fe}^{2+}+H_{2}O_{2}\to {Fe}^{3+}+{OH}^{•}+{OH}^{-}$$

(10) $${Fe}^{2+}+{OH}^{•}\to {Fe}^{3+}+{OH}^{-}$$

Although morphology influences the specific surface and number of active sites, as well as surface accessibility, both FeWO_4_ forms enable similar OH^•^ generation through this redox cycle, as confirmed by the similar drop in degradation activity upon IPA addition (39% vs. 35%). These results agree with previous studies. For example, Zhang et al. investigated photo-Fenton systems involving Fe_3_O_4_/α-FeOOH and FeWO_4_/GO [33]. They concluded that the photo-Fenton reactions involved are also controlled by OH^•^ radicals, even with other sources of charge (electrons or holes) [27].

It is worth emphasizing that the enhanced degradation observed at higher pH values cannot be directly ascribed to the increased generation of reactive oxygen species (ROS). The radical scavenging experiments (Figure 10) clearly demonstrated that hydroxyl radicals are the dominant species in both morphologies, independently of the solution pH. Thus, the improved photocatalytic activity under alkaline conditions is mainly related to electrostatic effects between the negatively charged catalyst surface (pH > pH_pzc_) and the cationic MB molecules, which favor adsorption and subsequent reaction, rather than to a higher intrinsic ROS yield.

### 2.7. Photocatalysis Mechanism Based on Photoelectrochemical Investigations

#### 2.7.1. Transient Photocurrent Response

**Figure 11:**
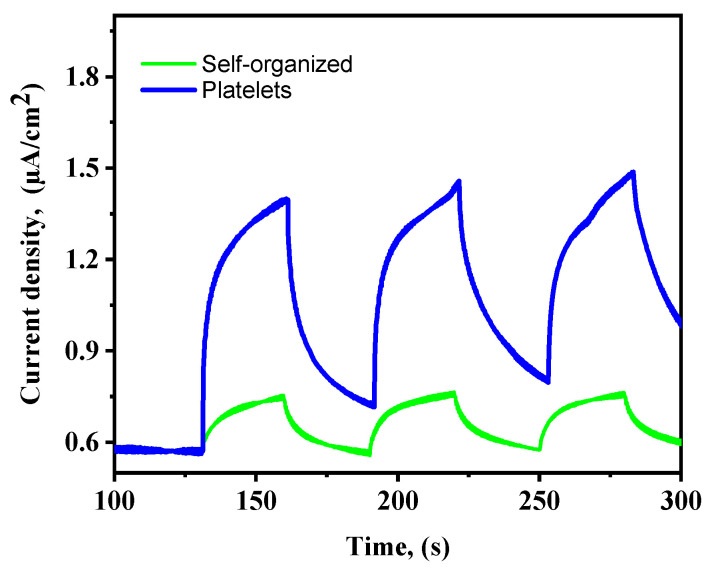
The transient photocurrent curves of self-organized and platelet-shaped FeWO_4_.

Transient photocurrent measurements were conducted under chopped wavelength illumination (390 nm) at an applied potential of +0.5 V vs. Ag/AgCl, optimized values to evaluate the generation and separation of photogenerated charge carriers. Figure 11 presents the current density versus time for the FeWO_4_ electrodes. The current density increased upon illumination and rapidly decreased when the light was turned off for both samples. The response was stable over the multiple light on/off cycles shown, indicating good photostability under these conditions and confirming its ability to generate hole–electron pairs. A significant difference in the photocurrent intensity was observed between the two morphologies. The platelet-shaped FeWO_4_ consistently generated a much higher photocurrent density, reaching peak values of approximately 1.48 µA/cm under illumination at this potential. By contrast, the self-organized FeWO_4_ achieved a peak photocurrent density of only about 0.78 µAcm^−1^. Beyond size effect, this difference is primarily due to the way the two samples are structured: the individual platelets possess twice the specific surface area of the self-organized ones, providing a larger surface for light absorption. Howover, the self-organized structures contains interstices and cavities which, through internal light reflection, reduce photoactivation. As reported in the literature, a higher photocurrent density generally implies more efficient generation and separation of electron–hole pairs, along with more effective transport of these carriers to the catalyst/electrolyte interface where they participate in redox reactions. Accordingly, based on their current density, platelets demonstrated enhanced photocatalytic activity in both the presence and absence of H_2_O_2_, compared to the self-organized ferberite.

In general, photocurrent measurement assesses the performance of a thin-film electrode under the influence of an external voltage bias. This setup favors materials that exhibit efficient long-range charge transport through the film to the collector. In this system, catalytic performance is governed by other factors owing to the use of isolated particles. This includes the conversion of reactant adsorption onto the particle surface and charge separation at the local surface level. The high photocurrent of the platelets demonstrates their catalytic activity when used as electrodes. Nevertheless, this parameter alone does not fully predict their performance in a suspended photocatalytic system, particularly since the self-organized material, while exhibiting lower current densities, shows a superior adsorption capacity.

#### 2.7.2. Mott–Schottky Analysis

**Figure 12:**
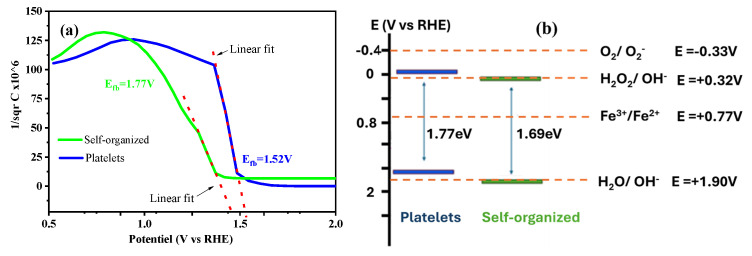
(**a**) Mott–Schottky plots (1/*C^2^* vs. *V*) of both FeWO_4_ electrodes measured at 300 Hz. (**b**) Energy band diagram with VB and CB edge positions in the NHE scale with the key ROS redox potentials involved in photodegradation mechanism.

To further investigate the semiconductor properties of the synthesized FeWO_4_ materials, Mott–Schottky analysis was carried out. Figure 12a displays the Mott–Schottky plots, i.e., 1/*C*^2^ vs. *V*, where *C* is the space charge capacitance and *V* is the applied potential. Both self-organized and platelets-like FeWO_4_ electrodes were evaluated at a frequency of 300 Hz. They exhibit negative slopes in the linear region of plots, which is indicative of *p*-type semiconductor behavior [60].

The flat-band potential (*V*_fb_) was determined by extrapolating the linear portion of the 1/*C*^2^ vs. *V* plot to the potential axis (i.e., where 1/*C*^2^ = 0). These experimentally determined *V*_fb_ values, combined with the optical band gap energies (1.69 eV for self-organized and 1.77 eV for platelet-shaped FeWO_4_), were used to calculate the conduction band (*E*_CB_) and valence band (*E*_VB_) edge position, as illustrated in Figure 12b.

For a *p*-type semiconductor, the Fermi level (EF) is close to *E*_VB_. Equation (11) is assumed as a common approximation for *p*-type material; the E_VB_ for self-organized FeWO_4_ is estimated at +1.97 V vs. NHE, and for plates at +1.72 V vs. NHE. Consequently, the *E*_CB_ positions (Equation (12)) are calculated to be +0.28 V vs. NHE for self-organized FeWO_4_ and −0.05 V vs. NHE for platelets-like FeWO_4_.

(11) $$E_{VB}=V_{fb}+0.2\;V$$

(12) $$E_{CB}=E_{VB}-E_{g}$$

The resulting band structure diagram, shown in Figure 12b, also includes the redox potentials of key ROS involved in the degradation mechanism. It provides a useful framework for interpreting the photocatalytic behavior of FeWO_4_. By correlating these energy levels with the results of radical scanvenging experiments, a coherent and plausible MB degradation mechanism can be proposed, as described in the following section.

#### 2.7.3. Photo-Fenton Mechanism and OH^•^ Generation Pathways in FeWO_4_/H_2_O_2_ System

The degradation mechanism in the FeWO_4_/H_2_O_2_ system under light irradiation proceeds mainly through the generation of hydroxyl radicals (OH^•^), as confirmed by scavenger experiments. Four potential pathways for OH^•^ production can be considered, each involving distinct redox processes. Their feasibility is discussed below based on the electronic band positions of FeWO_4_ and thermodynamic considerations.

- Reduction of H_2_O_2_ by Photogenerated Electrons

(13) $$H_{2}O_{2}+e^{-}\to {OH}^{•}+{OH}^{-}\;(E^{0}=+0.32\;V\;\mathrm{vs}.\;\mathrm{NHE})$$

This is the most plausible and dominant route. Both FeWO_4_ morphologies posses conduction band (CB) potentials more negative than +0.32 V (i.e., +0.28 V for self-organized and −0.05 V for platelets), making this reduction thermodynamically favorable. Therefore, photogenerated electrons can efficiently reduce H_2_O_2_ to *OH^•^*. This is consistent with trapping experiments showing that ^•^OH radicals are the primary reactive species involved in pollutant degradation.

b. Oxidation of OH^−^ by Photogenerated Holes

(14) $${OH}^{-}+h_{VB}^{+}\to {OH}^{•}\;(E^{0}=+1.90\;V\;\mathrm{vs}.\;\mathrm{NHE})$$

This pathway is only possible for the self-organized FeWO_4_, whose valence band (VB) lies at +1.97 V vs. NHE, slightly above the required potential. In contrast, the platelet morphology, with a VB at +1.72 V, cannot oxidize hydroxide to OH^•^. Thus, OH^•^ generation via hole-mediated OH^−^ oxidation is morphology-dependent and limited to the self-organized form.

c. Oxidation of H_2_O_2_ by Photogenerated Holes (Equation (13))

(15) $$H_{2}O_{2}+h_{VB}^{+}\to {OH}^{•}+H^{+}\;(E^{0}\approx +2.31\;V\;\mathrm{vs}.\;\mathrm{NHE})$$

Neither morphology has a VB potential high enough to drive this reaction. Therefore, this pathway is thermodynamically inaccessible under the given conditions.

d. Classical Fenton Reaction via Fe^2+^/Fe^3+^ Cycling

(16) $${Fe}^{2+}+H_{2}O_{2}\to {Fe}^{3+}+{OH}^{•}+{OH}^{-}$$

FeWO_4_ inherently contains Fe^2+^ species that can participate in the conventional Fenton reaction with H_2_O_2_ to generate OH^•^. Moreover, the photogenerated electrons in the CB are sufficiently reducing to convert Fe^3+^ back to Fe^2+^:

(17) $${Fe}^{3+}+e^{-}\to {Fe}^{2+}\;(E^{0}=+0.77\;V\;\mathrm{vs}.\;\mathrm{NHE})$$

This redox cycle is thermodynamically favorable for both morphologies, reinforcing the self-sustained nature of OH^•^ production through both photo- and Fenton-type mechanisms.

Among the four possible pathways, pathway (1) (H_2_O_2_ reduction by electrons) is active in both morphologies and is likely dominant. Pathway (4) (classical Fenton reaction) also contributes, aided by photogenerated electron-driven Fe^3+^/Fe^2+^ cycling. Pathway (2) is only feasible for the self-organized material due to its more positive VB, but contributes marginally. Pathway (3) is ruled out for both.

Interestingly, although the self-organized FeWO_4_ exhibits a more positive VB potential theoretically compatible with OH^−^ oxidation, it shows lower photocatalytic performance than the platelet morphology. This is consistent with our scavenging experiments, which revealed no significant contribution of photogenerated holes to the degradation activity in either morphology. These observations confirm that electron-driven processes—particularly H_2_O_2_ reduction and Fe^3+^ regeneration—are the dominant pathways in the photo-Fenton mechanism of FeWO_4_.

## 3. Experimental Section

### 3.1. Ferberite Hydrothermally Synthesis

In the syntheses, all chemicals were used as received with no further purification (sodium tungstate dihydrate (Aldrich, St. Quentin Fallavier, France, 99.0%), iron sulfate heptahydrate (Aldrich, France, 99.5%), oxalic acid (Aldrich, France, 99.5%), and hydrochloric acid (Aldrich, France, 37.5%)). FeWO_4_ powders were prepared by a hydrothermally route, according to the following procedure: first, sodium tungstate dihydrate (12 mM) was diluted in 100 mL of water and stirred for 10 min at room temperature (26 °C); the pH was adjusted by adding a 3 M HCl solution until the pH was 1. At this stage, there was a change in color, the transparent solution became light green; while stirring, we added oxalic acid (25 mM) in solid form and 150 mL of water, and the solution became transparent again. Finally, 14 mM iron sulfate hephydrate was added and transferred to the autoclave, which was heated to 200 °C for 24 h and then cooled naturally; the precipitate was collected and washed with water several times. Finally, the precipitate was dried at 80 °C for 24 h. During the synthesis process, approximately 10% of the ferberite mass was lost during the washing and drying steps of the precipitate obtained after the hydrothermal treatment. After the treatment process, the recovered powder was ground for appropriate physicochemical characterization and the evaluation of the properties of interest. To investigate the influence of iron (II) sulfate addition during synthesis, two experimental conditions were employed: (i) the direct addition of solid iron (II) sulfate heptahydrate to the final solution and (ii) the prior dilution of iron (II) sulfate in water before addition. As a result, two distinct types of ferberite samples were obtained, herein referred to as self-organized and platelet morphologies, respectively.

### 3.2. Structural, Morphological, Chemical, Textural Properties and Optical Investigation

The FeWO_4_ powders with different morphologies were structurally investigated by an Empyrean Panalytical diffractometer, equipped with a Cu anticathode. Powder X-ray diffraction (XRD) patterns were collected in the classic θ-2θ mode, from 10° to 80° (step size 0.003°, scan speed 0.004°s^−1^). To obtain the lattice parameters, crystal size and crystallographic data, the XRD patterns were used and refined by employing the Material Analysis Using Diffraction (MAUD) software version 2.064 [61]. The minimization was carried out using the reliability factors as numerical criteria of the refinement quality calculations (Rwp, Rexp and Rwp/Rexp(S)). To represent the refined structures, Carine Crystallography software (version 3.1) was used for a three-dimensional representation of the ferberite, illustrating the coordination polyhedral, and bonds between tungsten, iron, and oxygen.

The morphologies of the different powders were observed by transmission electron microscopy (TEM) and electron diffraction (ED) characterization were carried out using a conventional 200 kV Tecnai microscope equipped with a LaB_6_ source. The chemical composition of ferberite was determined by electron dispersive spectroscopy (EDS). Cation concentrations were quantified using standards. The statistical study of the chemical homogeneity of the powder consisted of between 10 and 15 analyses on randomly selected grains. Surface morphology was studied using a GEMINI Zeiss SUPRA 40Vp scanning electron microscope (SEM). Observations were made in InLens mode at a working distance between 2 and 3 mm and at a voltage of 5 kV without any prior treatment.

To verify the formation of products and intermediates during the synthesis of ferberite, aliquots of the solution were collected at each stage of the synthesis methodology: the addition of hydrochloric acid (1), the addition of oxalic acid (2), and the addition of iron sulfate hephydrate (3) and analyzed by Fourier transform infrared spectroscopy (FTIR) and MET.

Nitrogen adsorption–desorption isotherms (N_2_) were measured at 77 K using a Quantachrome Autosorb iQ AG surface area and porosity analyzer. The Brunauer–Emmett–Teller (BET) method was used to calculate the specific surface area from these isotherms [40]. Specific surface area, pore size and total pore volume were determined from the desorption branch of the isotherm using the Barrett–Joyner–Halenda (BJH) method [48]. Before analysis, the samples were degassed under vacuum at 200 °C for 24 h to remove any possibly adsorbed species. Finally, the optical properties (absorption, reflectance and band gap) of the obtained ferberites were investigated by UV-Vis spectroscopy in normal and diffuse reflectance modes with respect to photoactivated applications. The measured UV-Vis spectra of different samples were recorded using a Shimadzu 2600 UV/Vis spectrophotometer with a 150 mm integrating sphere and using BaSO_4_ as the baseline reference. Measurements were made in the range of 250–800 nm at room temperature with a resolution of 0.08 nm. The background was determined using a calibrated reflectance standard with an accuracy of 0.005.

### 3.3. Photoelectrochemical (PEC) Measurements

Photoelectrochemical characterizations (Mott–Schottky plot and transient photocurrent responses) were conducted using an OrigaLys (OrigaLys ElectroChem SAS, Rillieux-la-Pape, France) potentiostat/galvanostat in a standard three-electrode configuration. Working electrodes (WE) were prepared by depositing the synthesized FeWO_4_ materials (self-organized and plateles) onto conductive indium tin oxide (ITO)-coated glass substrates (1 cm × 1 cm). A suspension of 5 mg of FeWO_4_ powder in 1 mL of ethanol was prepared by ultrasonication for 30 min. A controlled volume of this suspension (1 mL) was drop-cast onto the active area of the ITO substrate and dried at 80 °C for 180 min. The reference electrode (RE) and the counter electrode (CE) were Ag/AgCl and platinum foil, respectively. The PEC measurements were performed using a 0.1 M aqueous solution of KOH (pH 14) and Na_2_ SO_4_ (pH 2) Mott–Schottky plot and transient photocurrent responses measures, respectively.

Transient photocurrent responses were measured under chopped illumination conditions. The light source was a Revoler Instytut Fotonowy LED system, providing monochromatic light at a wavelength of (390 nm). Measurements were typically recorded at an applied potential of +0.5 V vs. Ag/AgCl. Mott–Schottky analysis was used to determine the flat-band potential (*V*_fb_) of the FeWO_4_ semiconductor electrodes (both for self-organized and platelets) for a *p*-type semiconductor. The Mott–Schottky relationship is given by Equation (18).

(18) $$\frac{1}{C^{2}}=\frac{2}{\varepsilon \varepsilon_{0}A^{2}eN_{A}}\left(V-V_{Fb}-\frac{k_{B}T}{e}\right)$$

where *ε* is the dielectric constant of the semiconductor, *ε*_0_ is the permittivity of free space, e is the elementary charge, *A* is the electrode area, *V* is the applied potential, *k*_B_ is the Boltzmann constant, and *T* is the absolute temperature. The flat-band potential (*V*_fb_) was determined from the intercept of the linear region of the 1/*C*^2^ vs. *V* plot.

Potentials measured against Ag/AgCl were converted to the Reversible Hydrogen Electrode (RHE) scale using the Nernst equation, shown in Equation (19):

(19) $$E_{RHE}=E_{Ag/Agcl}+0.197\;V+0.059\;pH$$

where *E*_Ag/AgCl_ is 0.197 V vs. NHE at 25 °C of the Na_2_ SO_4_ (pH 2) and KOH (pH 14) electrolytes.

### 3.4. Photocatalytic Action Evaluation

The photocatalytic performance of the synthesized FeWO_4_ materials (self-organized and platelets) was evaluated by monitoring the degradation of methylene blue (MB), a model organic pollutant, under simulated solar irradiation. It is common to find concentrations around 5 ppm for MB in the literature, as it is a practical experimental range that avoids spectral saturation and maintains the linearity and efficiency of the process [57,62]. The photocatalytic system adopted basically consists of a 250 mL quartz tube with a water-cooling system coupled under a 300 W xenon lamp to simulate solar radiation. The spectral width of radiation from Philips lamps (300 W) simulating solar radiation, with maximum intensity near the visible region (400–800 nm).

First, the adsorption–desorption equilibrium was established. In a typical experiment, 100 mg of the FeWO_4_ catalyst was dispersed in 100 mL of an aqueous methylene blue (MB) solution with a concentration of 5 ppm. The study was conducted without pH adjustment (initial pH ≈ 5), with sampling at 10 min intervals over a period of 60 min. Once equilibrium was reached, preliminary photocatalytic experiments were carried out at the same pH (5) for 210 min, with measurements taken every 30 min.

To investigate the role of reactive oxygen species (ROS) and possible photo-Fenton contributions, comparative experiments were conducted under identical conditions using (i) hydrolysis (control), (ii) FeWO_4_ alone, (iii) H_2_O_2_ (8 mM) alone, and (iv) a combined FeWO_4_/H_2_ O_2_ (8 mM) system. Additionally, the influence of pH on MB degradation was systematically evaluated by adjusting the initial solution pH to 3, 5, and 10 using dilute nitric acid (HNO_3_) or sodium hydroxide (NaOH). These values were selected based on the point of zero charge (pH_pzc_) of the catalyst. When H_2_O_2_ was used, it was added after reaching the adsorption–desorption equilibrium time.

Based on the methylene blue degradation results as a function of pH variation, a second study was performed to investigate the effect of H_2_O_2_ concentration. Three conditions were evaluated: no H_2_O_2_, 4 mM H_2_O_2_, and 8 mM H_2_O_2_. Subsequently, to quantify the degradation kinetics of methylene blue, the experimental data were fitted to pseudo-first-order and pseudo-second-order kinetic models, as appropriate. The apparent rate constants (k_1_ and k_2_) were then determined according to Equations (20) and (21), respectively.

(20) $$ln\frac{C}{C_{o}}=-kt$$

(21) $$\frac{1}{C}-\frac{1}{C_{o}}=k_{2}t$$

At predetermined time intervals during the irradiation, approximately 3 mL of the suspension were withdrawn, centrifuged at 13000 rpm for 30 min to remove catalyst particles, and the supernatant was analyzed. MB concentrations were determined by measuring the characteristic absorbance peak at λ_max_ = 665 nm using a Shimadzu UV-1800 UV–visible spectrophotometer with version 2.71 of the software. Degradation activity was expressed as C/C_0_, where C_0_ is the initial MB concentration and C is the MB concentration at irradiation time t.

To evaluate the stability and reusability of FeWO_4_ (platelets and self-organized), recycling tests were carried out for the photodegradation of MB under optimized conditions (pH 5, 4 mM H_2_O_2_, under visible light). The catalysts were tested over four consecutive cycles. After each cycle, they were separated from the reaction mixture by centrifugation, washed with double-distilled water and dried overnight in an oven at 80 °C before. Additionally, XRD and FTIR analyses are performed to confirm the structural stability of the samples.

## 4. Conclusions

Nanostructured ferberite was successfully synthesized via hydrothermal routes under acidic conditions. The resulting powders crystallized in a single-phase monoclinic structure, exhibiting high purity, good crystallinity, and a near-stoichiometric composition (Fe: 49%, W: 51%). The investigation of the formation mechanisms revealed that the physical form of the FeSO_4_ precursor, whether introduced as a solid or in diluted solution, plays a key role in directing crystal growth and morphology. This led to the formation of two distinct structures: isolated platelets and self-organized desert rose-like microstructures. Optical and electrochemical measurements confirmed the *p*-type semiconductor nature of both morphologies, with band gap energies of 1.69 eV and 1.77 eV for self-organized form and platelets, respectively, indicating their sensitivity to visible light radiation. Photocatalytic tests under simulated sunlight revealed high degradation activity via photo-Fenton processes. The presence of H_2_O_2_ significantly enhanced the degradation rate of MB, achieving complete removal within 30 min. Each morphology exhibited optimal performance under different pH conditions, with a notable synergistic effect between the catalysts and H_2_O_2_, especially in alkaline environments. While both morphologies were active, kinetic analysis revealed a pseudo-second order behavior for platelet-shaped FeWO_4_ consistent with its enhanced textural properties. In contrast, the self-organized microstructure followed a pseudo-first-order kinetic model. The combined analysis of radical scavenging experiments and band structure diagrams identified hydroxyl radicals as the only reactive oxygen species (ROS) responsible for MB degradation. These results demonstrate the potential of nanostructured FeWO_4_ as a sustainable and efficient photocatalyst for water purification applications.

## Figures and Tables

**Table 1:** Lattice parameters and crystallographic data of FeWO_4_ synthesized via hydrothermal method under acidic conditions using iron (II) sulfate heptahydrate as precursor.

Compound FeWO_4_	Self-Organized	Platelets
Space group	P2/c:1	
Cell parameters (Å)	a = 4.733	a = 4.731
	b = 5.710	b = 5.708
	c = 4.977	c = 4.976
Angle (degrees)	β = 90.334	β = 90.292
Density (g/cm^3^)	7.49	7.50
Crystal size (nm)	67.78	87.28
**Reliability factors**		
Rwp (%)	14.9	12.8
Rexp (%)	12.9	10.5
Rwp/Rexp(S)	1.16	1.22

**Table 2:** Comparison of photocatalytic performance of FeWO_4_ catalysts synthesized in this work and other representative reported FeWO_4_-based catalysts for MB degradation.

Synthesis Method	Morphology/ Composite	Volume (mL)	Mass Masse (mg)	Irradiation	[H_2_O_2_] (mM)	pH	Time (min)	MB Removal (%)	Reference
Hydrothermal	Platelets/ FeWO_4_	100	100	Simulated sunlight	4	5	30	94–100	This work
Hydrothermal	Self-organized /FeWO_4_	100	100	Simulated sunlight	4	5	30	92–99	This work
Hydrothermal	Nanorods/FeWO_4_	300	300	Visible light	66	5	60	~90	[22]
Microemulsion/ Hydrothermal	Nanorods/FeWO_4_	100	25	Visible light	–	5	120	75	[29]
Microemulsion	Ni–FeWO_4_	-	100	UV light	–	3	60	98	[24]
Co-precipitation-assisted hydrothermal	Nanosheets/FeWO_4_–GO (I-doped)	100	15–40	Sunlight	–	7	25	95	[30]
Hydrothermal	Nanofibers/ FeWO_4_	50	100	Visible light + ultrasound	2	7	14	99.5	[49]
Hydrothermal	NI */(30wt%-TiO_2_-250)	500	100	UV light	5	10	120	89.53	[56]
Coprecipitation	Spherical/ (NiO/Ag/TiO_2_)	80	2/4/8	UV light	–	NI	60	93.15	[57]
Precipitation/ Hydrothemal	Nanorods/ZnO-NR/ACF	100	50	UV light	–	6.7	120	99	[58]

* NI—not informed by the authors.

## Data Availability

Data will be made available on request.

## Supplementary Materials

The following supporting information can be downloaded at: https://www.mdpi.com/article/10.3390/molecules30194026/s1, Figure S1. Electronic diffraction pattern (TEM) of ferberite: Platelets (a) and self-organized (b) Section 2.5 dedicated to the optical properties and UV-vis diffuse reflectance. Figure S2. Corresponds to the UV-vis absorption spectra of MB solution (5 ppm) in presence of platelets ferberite morphology at pH 5; Figure S3. Concerns FTIR (a) and XRD (b) of powders after two cycles of photodegradation.
