# Supplementary material for: Insights of Nanostructured Ferberite as Photocatalyst, Growth Mechanism and Photodegradation Under H2O2-Assisted Sunlight
Source: Molecules. 2025 Oct 9;30(19):4026. doi: 10.3390/molecules30194026 (PMC12526424; doi:10.3390/molecules30194026)
Supplement: Supplementary file 1 [file molecules-30-04026-s001.zip › molecules-3783498-supplementary.pdf]

# Supplementary Materials

*Article*

# Insights of Nanostructured Ferberite as Photocatalyst, Growth Mechanism and Photodegradation under $H_2O_2$-assisted sunlight.

**Andarair Gomes dos Santos [1,2], Yassine Elaadssi [1], Virginie Chevallier [1], Christine Leroux [1], Andre Luis Lopes Moriyama [1,3], Madjid Arab [1,*]**

[1] Université de Toulon, Aix Marseille Univ, CNRS, IM2NP, Marseille, France,
email: yassine-elaadssi@etud.univ-tln.fr, cheva@univ-tln.fr, christin.leroux@wanadoo.fr, madjid.arab@univ-tln.fr
[2] Universidade Federal Rural do Semi-Árido, CCEN, Campus Mossoró-F. Mota, Costa e Silva, 59.625-900, Mossoró/RN, Brasil, email: andarair@ufersa.edu.br
[3] Universidade Federal do Rio Grande do Norte, Campus Universitário, L. Nova, 59072-970, Natal/RN, Brasil,
email: andre.moriyama@ufrn.br
*Correspondence: *madjid.arab@univ-tln.fr*

2.3.2 Nucleation and Growth Mechanism: Insights from TEM and FTIR Analyses

Microstructrural investigation from Electronic diffraction analysis (TEM)

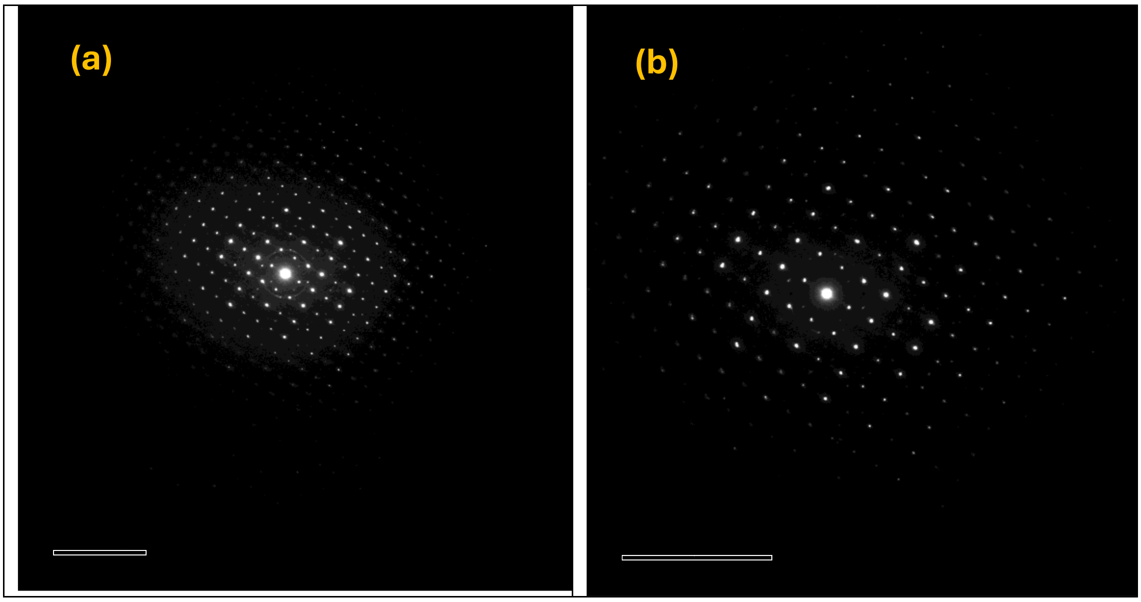


**Figure S1.** Electronic diffraction pattern (TEM) of ferberite: Platelets (a) and self-organized (b).

*2.5. Optical properties – UV-vis diffuse reflectance*

$$(\alpha h\nu) = B\left(h\nu - E_g\right)^{1/n} \qquad \text{Eq. S1}$$

where $\alpha$ is the absorption coefficient, h is Planck's constant ν the photon frequency, and n characterizes the type of electronic transition: n = 2 for direct allowed and n=1/2 for indirect allowed transitions. Eg was estimated by extrapolating the linear portion of the Tauc plot $(\alpha h\nu)^{1/n}$ versus hν to the x-axis ($(\alpha h\nu)^{1/n} = 0$). In view of this ongoing debate, both direct ($n = 2$) and indirect ($n = 1/2$) electronic transitions were considered in our analysis.

*2.6. Photocatalytic Activity of Ferberite with Self-organized and Platelets Morphologies*

2.6.1 Adsorption Studies

Figure S2 presents the evolution of the UV-Vis absorption spectra of MB over time in the supplementary Materials

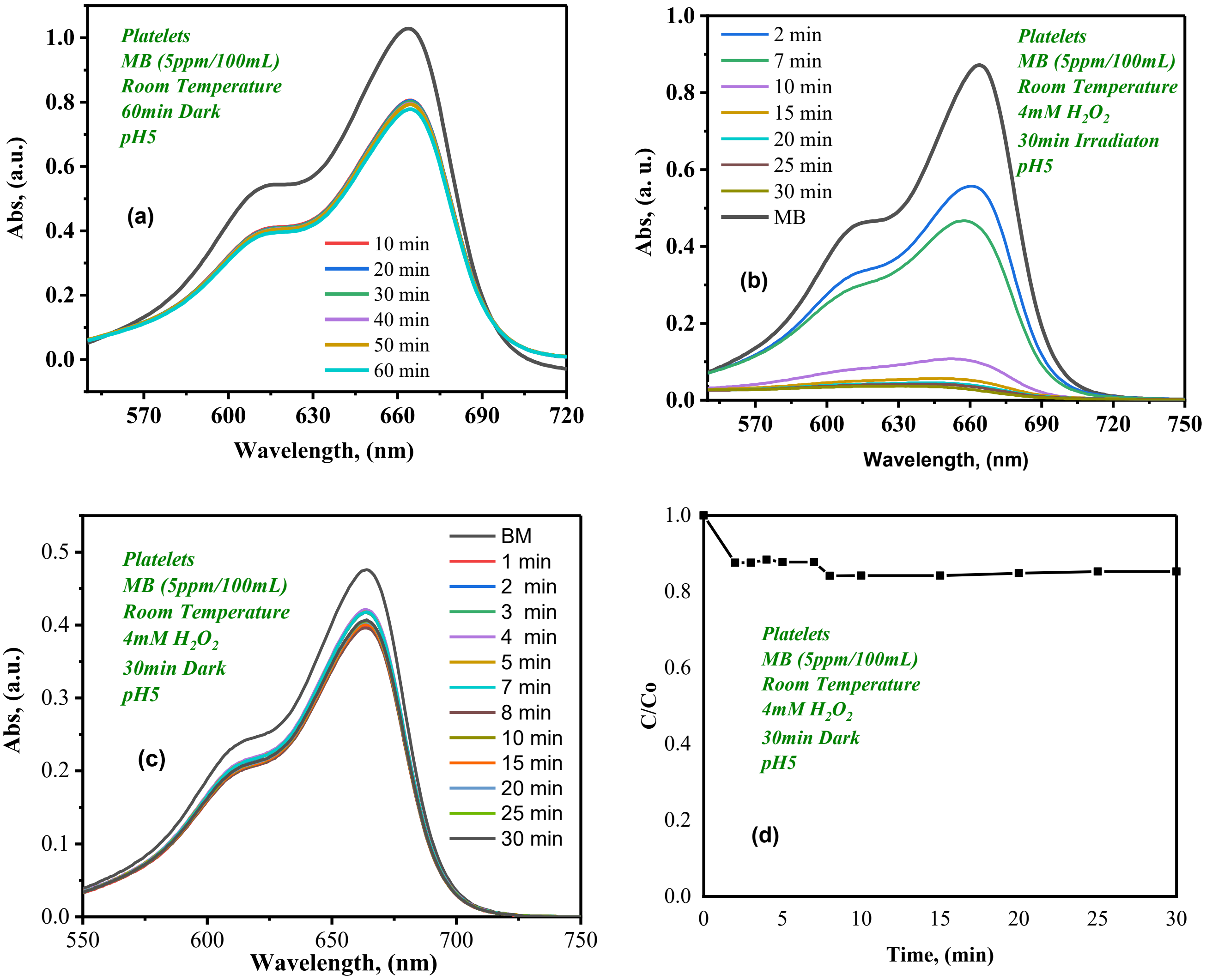


**Figure S2**. UV-vis absorption spectra of MB solution (5 ppm) in presence of platelets ferberite morphology at pH 5: (a) in the dark, (b) under sunlight irradiation with $H_2O_2$ assistant and (c) Fenton.

## h. Recyclability and stability

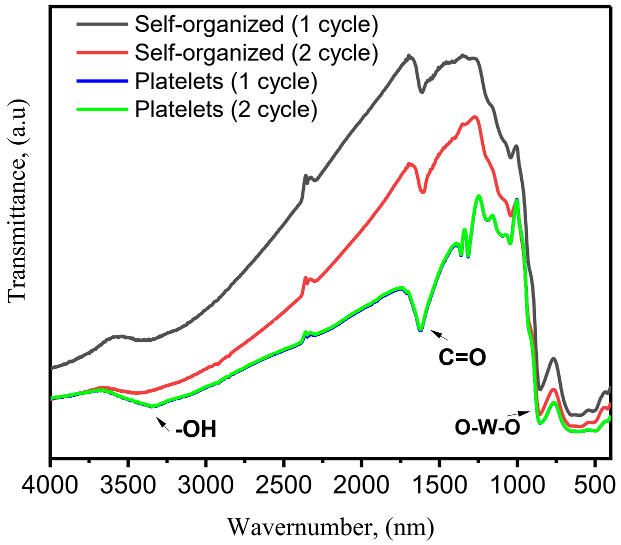


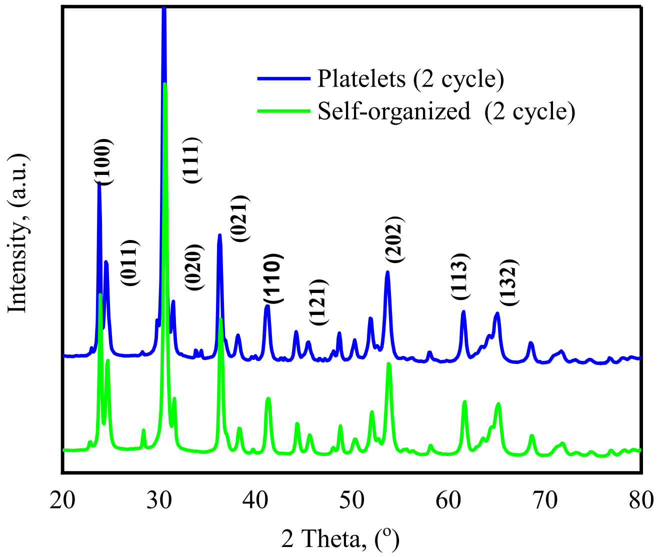


**Figure S3.** FTIR (a) and XRD (b) of powders after photodegradation (2 cycles).

Figure S3(a) shows the FTIR spectra recorded at 400-4000 $cm^{-1}$ for the synthesized $FeWO_4$ powders after two cycles of MB photocatalysis. The broad absorption bands observed around 3400 and 1600 $cm^{-1}$ are correlated with -OH stretching and bending distortion due to $H_2O$ absorption, respectively[1], [2]. The typical elongated band at 550 $cm^{-1}$ is related to bending vibrations due to Fe-O [1]. The tungstate category of $ABO_4$ in the 900-400 $cm^{-1}$ range exhibited characteristic stretching absorption bands. The deformation mode of the $WO_4$ tetrahedron was attributed to these shorter wavelength bands. However, the peaks at around 850 $cm^{-1}$, the broad absorption bands may be related to the bending vibrations and stretching of the W-O bonds in $WO_4$ [2]. Even after 2 cycles the profile practically does not change, especially for ferberite with platelets morphology. hile Figure S3 (b) shows the diffractograms also after 2 cycles, demonstrating that there was no structural change in both morphologies (self-organized and platelets).

[1] M. Irfan *et al.*, « The Fabrication of Halogen-Doped $FeWO_4$ Heterostructure Anchored over Graphene Oxide Nanosheets for the Sunlight-Driven Photocatalytic Degradation of Methylene Blue Dye », *Molecules*, vol. 28, nº 20, p. 7022, oct. 2023, doi: 10.3390/molecules28207022.
[2] S. M. Abdelbasir, A. M. Elseman, F. A. Harraz, Y. M. Z. Ahmed, S. M. El-Sheikh, et M. M. Rashad, « Superior UV-light photocatalysts of nano-crystalline (Ni or Co) $FeWO_4$ : structure, optical characterization and synthesis by a microemulsion method », *New J. Chem.*, vol. 45, nº 6, p. 3150-3159, 2021, doi: 10.1039/D0NJ05431C.
